# Evaluation of a video to promote HIV testing in sexual minorities

**DOI:** 10.1590/0034-7167-2023-0320

**Published:** 2024-11-22

**Authors:** Francisco Javier Báez Hernández, Vianet Nava Navarro, Miguel Angel Zenteno López, Víctor Manuel Blanco Álvarez, Arelia Morales, Pedro Trejo Hernández

**Affiliations:** I Benemérita Universidad Autónoma de Puebla. Puebla, Mexico

**Keywords:** Instructional Film and Video, Validation Study, Audiovisual Resources, HIV Testing, Sexual and Gender Minorities, Peliculas y Videos Educativos, Estudio de validación, Recursos Audiovisuales, Prueba de VIH, Minorias Sexuales y de Genero

## Abstract

**Objective:**

To design and evaluate an educational video aimed at promoting HIV testing in gay men from the theoretical perspective of the Nola J. Pender Health Promotion Model.

**Methods:**

The design comprised five steps: 1.- Literature search; 2.- Formulation of the educational objective; 3.- Drafting of the script and location of the information in the theoretical components; 4.- Production; and 5.- Evaluation by experts and the target population.

**Results:**

The video “Living Without Fear” was produced, which presents the dilemma faced by gay men before taking a HIV test. The content validity index was 0.85, which indicated that the information was adequate and acceptable for promoting the rapid HIV test.

**Final Considerations:**

The results contribute to the scientific evidence aimed at promoting healthy behavior. In addition, the video was shown to be an acceptable educational tool.

## RESUMEN

**Objetivo:**

Evaluar el contenido de un video educativo dirigido hacia la promoción de la prueba de VIH en varones homosexuales bajo la perspectiva teórica del Modelo de Promoción de la Salud de Nola J. Pender.

**Métodos:**

El diseño consistió en cinco pasos: 1.- Búsqueda de literatura; 2.- Elaboración del objetivo educativo; 3.- Construcción del guión y ubicación de la información en los componentes teóricos; 4.- Producción; 5.- Evaluación por expertos y población objetivo.

**Resultados:**

Se produjo el video “*Vivir sin Miedo*”, que presenta el dilema que tiene un varon homosexual, ante la realización de la prueba de VIH. El índice de validez de contenido obtuvo un valor de .85, lo que indica que la información es adecuada y aceptable, para la promoción de la prueba rápida de VIH.

**Conclusiones:**

Los resultados contribuyen a la evidencia científica dirigida a la promoción de conductas saludables, además se muestra como una herramienta educativa aceptable.

## INTRODUCTION

Human immunodeficiency virus (HIV) is a lentivirus from the *Retroviridae* family that directly affects CD4^+^ lymphocytes, the cells coordinating the defense mechanisms of humans^(1)^. In 2021, 38.4 million people worldwide were living with HIV^(2)^.

An estimated 341,313 people have HIV in Mexico^(3)^. The situation is similar in the Mexican state of Puebla, with 521 cases reported in 2022; the most affected population was gay men^(4)^.

Gay men are often subject to discrimination and stigma^(5-7)^, which are associated with structural barriers that become evident in the organization of healthcare institutions and the care received^(8-9)^. This situation aggravates additional concerns about privacy, the low perceived risk of contracting HIV, a lack of understanding of sexual health, and an inability to find places for undergoing rapid HIV testing^(10-12)^.

In this regard, the scientific literature has shown that gay men who are in the habit of undergoing rapid HIV testing have improved sexual behavior, due to increased self-efficacy, and, by undergoing this screening, exhibit better self-assessment^(13-15)^.

The strategies used to promote HIV testing include educational videos^(16)^, which are characterized by dramatic and documentary-based contents reflecting the actual lived-in experiences of gay men. These videos are based on more than one theory of change, have durations of between 1 and 83 minutes, and have exhibited favorable short-term effects on perceived barriers and risky behavior and improved understanding, self-efficacy, and acceptance of HIV testing in the population in question^(16-19)^.

The design and evaluation of the educational videos developed to prevent sexually transmitted infections have been found to be important for providing scientific evidence, as well as for ensuring content consistency, acceptability, and likely effectiveness in the target population^(20-22)^.

For this reason, the design and assessment of a video based on the Health Promotion Model theory^(23)^ becomes relevant. This theory considers health behavior to be the result of cognitive processes of attention, retention, reproduction, and motivation, which are associated with increased learning and skills related to preventive actions. In this model, individual characteristics and experiences are always central. This becomes evident when considering the creation of a video as an educational tool that can raise awareness of the importance of screening as a self-care method and that considers the needs and expectations of gay men.

Accordingly, and given the scant evidence from Mexico, with just four videos promoting HIV testing on the official webpage of the National Center for the Prevention and Control of HIV and AIDS^(24)^, we proposed the following:

## OBJECTIVE

To design and evaluate an educational video aimed at promoting HIV testing in gay men under the theoretical perspective of the Nola J. Pender Health Promotion Model^(23)^.

## METHODS

### Ethical aspects

This research study adhered to national and international bioethics principles and to the Law on the Protection of Personal Data Held by Private Parties^(25)^ and the General Health Law of Mexico^(26)^. Informed consent was obtained from all participants, and the study was registered with a bioethics and research committee (SIEP/046/2021).

### Study type

The study design comprised five steps based on similar studies^(20-22,27)^, summarized as: 1.- Literature review; 2.- Formulation of the objective of the educational video; 3.- Drafting of the script and placement of the information in the theoretical components of the Health Promotion Model^(23)^; 4.- Production; and 5.- Assessment of the content by experts and the target population.

### Study stages

The first step comprised an integrative literature review, following the five steps proposed by Whittemore (2005) ^(28)^: 1.- Problem identification; 2.- Literature search; 3.- Assessment of study quality; 4.- Data analysis; and 5.- Presentation of results. The question posed was, What is the best evidence available on the interventions used to promote rapid HIV testing in gay men? The following inclusion criteria were applied: randomized clinical trials in English, Spanish, or Portuguese, without restriction on year of publication, and full-text articles only. Two scientific databases were searched, PubMed and ProQuest, using Health Sciences Descriptors (DeCS) and Boolean operators (AND, OR, NOT) to construct search strings (“intervention” OR “random clinical trial” OR “clinical trial” AND “hiv test” AND “msm” OR “gay” NOT “women”). The exclusion criteria were as follows: meta-analyses or meta-syntheses, gray literature, opinions, editorials, and clinical cases or reports. Duplicate studies were eliminated from the database to avoid data duplication.

As well as using Mendeley reference manager software, we constructed a data analysis matrix containing data related to the objective, theory, population, method, intervention characteristics, results, and references in order to identify the strategies and cinematographic techniques with the best results.

In the second step, we formulated the objective of the educational video in order to better conceptualize and plan the creation of the audiovisual product. This step helped us to define the type of educational video, as well as the elements best suiting its purpose and the characteristics of the target audience (age group, medium, and execution feasibility). For its creation, we used Benjamin Bloom’s taxonomy^(29)^, followed by a description of the area of achievement (content or topic), the indicator (criteria), and the strategies or considerations for achieving it.

The third step was divided into two phases. The first involved the drafting of the script with the support of a multidisciplinary team. The team included specialists in social communications, who were chosen due to their experience in the production of health-related videos, and specialists in the care of people diagnosed with HIV. All were invited to participate by email. The drafting of the script adhered to that reported in related studies and in the information contained in the Mexican National Center for the Prevention and Control of HIV and AIDS and to the suggestions of a group of self-identified gay male youths considered community leaders. These young men were first explained the objective of the video creation to motivate their participation. They were invited and selected in a joint manner by a key participant recruiter and the researchers.

The second phase comprised placement of the information in the script based on the theoretical components of the Health Promotion Model^(23)^. This was conducted via the creation of a matrix relating theoretical concepts to the components of the educational video, which was organized in the following manner: Health Promotion Model, Content, Images, and Text^(22)^.

The fourth step was the production of the educational video. The video was produced in a public building and with the corresponding pre-approval. Most actors, including the main character, were members of the LGBTIQ+ community. The audio was recorded in a quiet location with suitable acoustics. Editing was conducted with CapCut software. The initial version was converted into MP4 format.

Finally, the fifth step comprised assessment of the content by six researchers considered experts due to their career and publications on sexual health and HIV. The researchers assessed the following aspects: objective (focused on care, demystification, prevention, information, and ideas concerning HIV), language (message clarity), relevance (video efficacy, appropriateness, and consistency), content (clear, objective, and scientifically accurate story based on evidence), and design (attractive presentation and adequate length) ^(30)^. A Likert scale was used to score these aspects from 1 to 5 (1=completely disagree; 2=disagree; 3=neutral; 4=agree; 5=completely agree). The data were processed in Microsoft Excel, which was used to obtain the content validity index (CVI) ^(31)^. The instrument content was considered adequate with a CVI score equal to or higher than 0.80.

### Analysis of results and statistics

In addition, we applied surveys of acceptability and preliminary efficacy^(18)^, which were adapted for this study for a group of 18 gay men. These individuals were chosen and selected by two participant recruiters belonging to the LGBTIQ+ community who began the snowball sampling. The participants were given a week to respond to the following questions: “How much did you like the video ‘Living Without Fear’?”; “How much would you recommend the video ‘Living Without Fear’ to a friend?”; “Was it sometimes difficult to understand what was happening in the video?”; “Did your mind sometimes wander while watching the video?”; “Were the events in the story depicted in the video relevant for your daily life?”; “Do you think that the information provided on certain topics was inaccurate or misleading?”; “Did you understand the feelings and emotions of the main character”?; “In the future, do you plan to undergo regular HIV testing?”; and “Would you like to schedule an appointment for a HIV test?”. The assessment was satisfactory when most responses indicated good understanding and acceptability of the video content. All of these analyses were performed using Statistical Package for the Social Sciences (SPSS version 26.0), which provided the frequencies and percentages for each question.

## RESULTS

The literature review identified randomized trials with the following characteristics: use of electronic social media networks, web pages, and videos. The latter were typically short and provided simple information on HIV and promoted testing. In addition, they used behavioral theories such as community-based participatory research, the social cognitive framework, the health belief model, and integrated behavior model. The strategies used included the inclusion of peers and community leaders, as well as relatable actors, with the use of the vernacular familiar to the target population. In addition, they considered sociocultural and economic barriers, as well as the motives and significant experiences of the participants, not only when they are undergoing the rapid HIV test, but also when they are sexually active.

After the literature analysis, the overall objective was established by selecting an action verb from the affective domain of Bloom’s taxonomy^(29)^. This domain focuses on individuals’ observable behaviors by including learning associated with feelings, emotions, and attitudes. The objective was to motivate gay men to take the rapid HIV test by considering their perceptions, influences, beliefs, and experiences that promote or limit health-promoting behavior (Chart 1).

**Chart 1:** Objective of the educational video

*To promote rapid HIV testing in young men in Puebla via the narrative testimony of a successful case and based on the theoretical perspective of Murdaugh, Parsons, and Pender (2018).*

Based on the previous point, we developed the script for a video entitled “Living Without Fear” (“*Vivir sin Miedo*”). The script considered the opinions of a group of young gay men, as well as certain neuro-linguistic programming and sensory marketing tools, given that perception is influenced by beliefs and values. For this reason, communication is not limited to written or spoken language but also involves multiple tools related to feelings and memories that are embedded in an individual’s conscious or unconscious mind and that can help to strengthen the message^(32-33)^. Together, the efficacy of information reception increases with access to all senses, enabling the more complete and interactive persuasion of individuals^(34-35)^.

Given the above, we decided to use narrative testimony, a communication technique that is based on a phenomenological approach to appreciating experiences. This technique weighs up the facts based on how they are lived and felt and how they influence and affect individuals, thereby facilitating the understanding of values, situations, history, and lived experiences^(36)^. Once the script was obtained, this approach allowed us to place all of the information in the components of the Health Promotion Model^(32)^ (Chart 2).

**Chart 2:** Relationship of the Health Promotion Model with the components of the educational video

Health Promotion Model	Content	Image	Text
-----	Opening credits	Video title	“Living Without Fear”
Health-Promoting Behavior	Virus that attacks the body’s defenses and can be controlled.	Eduardo looks at the device screen that will give him the test result. He looks up at the camera.	My name is Eduardo and, a few days ago, I had an experience that has changed me forever.
Situational Influences	By contact with body fluids, due to unprotected sex, and sharing infected needles. Sometimes during pregnancy, birth, or breastfeeding.	Eduardo receives WhatsApp messages from his sexual partner describing the signs and symptoms of HIV infection.	After being with Gabo, the messages that he sent me are those that nobody wants to receive - he was having a hard time.
Perceived Barriers to Action	It is not spread by insect bites, saliva, tears, or sweat or by hugging, holding hands, using the same toilet, sharing plates, or “social kissing”.	Eduardo is clearly anxious and is unable to sleep in his room.	Something was wrong and it wouldn’t stop going around my head. So much is said about HIV that I don’t know what to believe: that you can catch it by using the same toilet or sharing plates. My head is going to explode!
Situational Influences	The myths of HIV, that only certain groups can contract the virus, prejudices anyone who undergoes the test and suggest that it is some form of divine punishment.	Eduardo, checking Grindr, finds the advertisement for rapid tests in BUAP Clinic.	Only we understand and support each other. What is this? BUAP performs rapid tests and the center is close to here! Hmmm. I don’t know though. There are so many haters and I can’t handle that.
Self-Efficacy Personal Sociocultural Factors Situational Influences	The rapid test is effective if it is performed between 18 and 90 days after exposure.	Eduardo decides to attend the center to be tested. Eduardo finds a warm and friendly atmosphere (polite men and women). A girl shows him to the consulting room. A smiling couple leaves the consulting room.	I’ve made up my mind: if I don’t take care of myself, how can I take care of Gabo? What a cool place. Nobody’s giving me bad vibes. Wow, they’re folks like me. Friendly. Here I go.
Situational Influences Health-Promoting Behavior	Your finger is pricked to collect a drop of blood, from which a reagent gives a positive or negative result.	Eduardo enters the consulting room and is received by a health care professional. Eduardo undergoes the test.	The nursing staff treating me are so discreet, clean, and laidback. Hmmm, kinda scary but, come on, it’s just a prick.
Perceived Barriers to Action Health-Promoting Behavior Commitment to Plan of Action	You have the result in less than 10 minutes. It is 98% accurate. You do not need to fast beforehand. The consultation is discreet, anonymous, and confidential. The result gives you peace of mind, control over your health, and confidence to conduct your sex life in a full and responsible way.	Eduardo’s test result is negative and he receives more information (on the test and HIV) Transition. Eduardo accompanies his partner to undergo the test in the clinic.	Cool! In 10 minutes, all doubts were relieved: they explained that the result if 98% accurate and that no preparation is required. It is discreet, anonymous, and confidential. Now I can fully enjoy my sex life. Well, Gabo and I can. This is loving yourself... let’s go!
--------	Closing credits	Credits and logos	Black screen

Video production lasted 1 week. During this time, the following effects were added, in addition to Spanish subtitles: flashback, black and white shots, color transitions, slow motion shots, and tilt down shots toward the eyes of the main actor. The final result was a video with a duration of 1 minute 49 seconds. Subsequently, the content was assessed by six experts in sexual health and HIV (physicians, nursing staff, psychologists, anthropologists, and pharmacobiological chemist). A CVI of 0.85 was obtained, indicating that the information used was considered adequate and appropriate for promoting rapid HIV testing in gay men (Table 1). However, we first considered the recommendations of the judges in the domain of the objective in terms of making the risk factors more explicit, such as unprotected sexual activity, and how the creation of friendly spaces without sexual preference-related stigma is one of the strategies for preventing HIV.

**Table 1:** Assessment by judges

Domain	Question	CVI
1 - Objective	Does it make viewers think about the care that gay men should take in their sexual relations?	0.73
	Does it help to demystify the topic of HIV?	0.83
	Does it promote a change in behavior and attitude toward the lack of prevention?	0.83
	Does it clarify information on the rapid HIV test?	0.70
	Does it mitigate prejudices concerning HIV?	0.80
2 - Language	Is the presented content understandable?	0.80
	Is the content understandable due to the use of familiar words and simple definitions?	0.90
	Is the content understandable due to good grammatical agreement?	0.90
3 - Relevance	Is it relevant as a health education tool?	0.93
	It is suitable for gay men?	0.93
	Does it offer an opportunity to better understand the rapid HIV test?	0.90
	Does it focus on the key aspect, the performance of the rapid HIV test?	0.93
4 - Content	Is it able to clarify doubts on the rapid HIV test?	0.76
	Are the messages presented in a clear and objective manner?	0.80
	Is the information presented scientifically correct?	0.93
	Is the content sufficient to achieve the objective?	0.70
	Is there a logical sequence to the presented information?	0.86
5 - Design	Is the video presentation attractive and well organized?	0.96
	Is the video the appropriate length?	0.96
Overall		0.85

*CVI, content validity index.*

Finally, the video was shown to 18 gay men, mainly university students, with a mean age of 20.1 ± 1.25 years (range, 18-21 years). The preliminary acceptability and efficacy surveys proposed by del Rio-González et al. (2021) ^(18)^ revealed a high percentage of acceptance and recommendation among the people who watched the video “Living Without Fear”.

Regarding the narrative engagement, most of those surveyed found it easy to understand what had happened in the video and felt that the events in the story were relevant for their lives. For the counterarguing, most of those surveyed considered the information shown in the video to be accurate and trustworthy and identified with the main character’s feelings or emotions. In terms of HIV testing, all respondents stated that, after viewing the video, they planned to take the test (Table 2).

**Table 2:** Acceptability and efficacy of the video “Living Without Fear”

Dimension	Question	Response type							
		A little		Average		A lot		Quite a lot	
		%	n	%	n	%	n	%	n
Acceptability	How much did you like the video “Living Without Fear”?	5.6	1	33.3	6	33.3	6	27.8	5
	How much would you recommend the video “Living Without Fear” to a friend?	0.0	0.0	27.8	5	38.9	7	33.3	6
**Dimension**	**Question**	**None**		**A little**		**A lot**		**Quite a lot**	
		**%**	**n**	**%**	**n**	**%**	**n**	**%**	**n**
Counterarguing	Do you think that the information provided on certain topics was inaccurate or misleading?	61.1	11	38.9	7	0.0	0	0.0	0
Identification with the main character	Did you understand the feelings and emotions of the main character?	0.0	0	5.5	1	66.7	12	27.8	5
**Dimension**	**Question**	**Completely disagree**		**Disagree**		**Agree**		**Completely agree**	
		**%**	**n**	**%**	**n**	**%**	**n**	**%**	**n**
Narrative engagement	Was it sometimes difficult to understand what was happening in the video?	44.5	8	33.3	6	22.2	4	0.0	0
	Did your mind sometimes wander while watching the video?	50.0	9	50.0	9	0.0	0	0.0	0
	Were the events in the story depicted in the video relevant for your daily life?	0.0	0	16.7	3	55.6	10	27.7	5
Intention to undergo the HIV test	In the future, do you plan to undergo regular HIV testing?	0.0	0	0.0	0	38.9	7	61.1	11
	Would you like to schedule an appointment for a HIV test?	0.0	0	11.2	2	44.4	8	44.4	8

*n - number of participants; % - percentage.*

## DISCUSSION

The present study reported the design and assessment process for an educational video aimed at promoting HIV testing in gay men. This screening action reflects a state of awareness after risk exposure, encouraging both self-care and the care of others in the case of infection, under the theoretical perspective of the Health Promotion Model^(23)^. Our approach was based on an analysis of the scientific literature, similar to that performed by other studies^(20,22)^, which showed audiovisual resources (videos) to be a promising tool for promoting healthy sexual behavior. This video could have major positive effects on Mexican gay men if it is disseminated within gay hook-up apps.

The objective of the video “Living Without Fear” was established in a similar manner to that performed in Brazil^(27)^ in which the CTM3 method was used. We first conceptualized the product that we wished to obtain. This permitted us to reflect on what Bloom refers to in his theory of learning^(29)^, specifically the affective input characteristics that motivate individuals. These characteristics can drive them to achieve learning outcomes and therefore potentially inspire changes in attitudes and behaviors related to self-care among gay men.

The script drafting and video production were again similar to those of other studies performed in Brazil^(21-22)^. Throughout, we considered the opinions and lived experiences of gay men, as in other studies from Mexico^(5)^ and Brazil^(12)^, as well as prejudices in the healthcare needs of gay men, a situation that triggers fear and self-exclusion from medical care.

This last reflection allowed us to incorporate into the script the neuro-linguistic programming tool visual anchoring^(32)^, which was associated with the emotional experience of gay men taking the HIV test for the first time. This tool was enhanced by sensory marketing strategies, which were key elements in the testimonial narrative. These strategies assumed that experience is a meaningful unit and gave voice to those who have been excluded, made invisible, and marginalized by society^(36)^.

Due to the elements contained in the script, the video “Living Without Fear” aligns with the Interpersonal Influences concept within the theoretical components of the Health Promotion Model^(23)^. This is because it is a primary source that aims to convince gay men to adopt health behaviors under the assumption that individuals, in all of their biopsychosocial complexity, can progressively change over time.

In this particular case, we operationalized the above aspect in the storyboard through the interrelation of personal sociocultural factors, situational influences, benefits, barriers, and perceived self-efficacy by using relatable actors and language and locations recognized by the target population. In addition, the video showed the conflict that they face when considering the rapid HIV test and how they overcome it, namely by finding an accessible, discreet, and respectful place. Taken together, these factors achieve a commitment to an action plan and, consequently, health-promoting behavior, specifically the acceptance and performance of the rapid HIV test. This situation acts not only as a basis for care actions, but also generates a bridge between theory and Advanced Practice Nursing, located at the primary care level.

In relation to the assessment process, the findings are in line with those of other studies addressing issues of sexually transmitted diseases in social work^(30)^ and the promotion of HPV vaccination^(21)^ and understanding^(22)^, with adequate scores in the domains of language, relevance, content, and design. The results show that the video “Living Without Fear” is an accepted and recommended educational tool for the prevention and the promotion of rapid HIV testing. In addition, the proposal was enriched and improved by the opinions of the judges in order to achieve the proposed objective.

As for the acceptability and effectiveness surveys, the results are in line with those obtained in Colombia^(18)^, which showed good acceptability by the population of gay men, mainly through self-identification with the main character and his counterarguing and intentionality to take the HIV test. However, the results for narrative engagement were higher for our video. This may be due to the short length of the film, as well as the incorporation of the main character’s verbal and non-verbal actions and language, which could have been identified as part of the daily life of the key population. This result highlights the importance of always considering the participation and opinions of gay men when designing health educational materials.

### Study limitations

As limitations, we note that the video was made based on the perceptions and tastes of a group of gay men from the city of Puebla. Accordingly, they cannot be generalized to the entire LGTBQ+ community in Mexico because tastes constantly change and are limited to the culture and social context where the study was conducted. In addition, knowledge about HIV is not static, and continuous updating of the information provided is required. Nonetheless, we aimed to reduce these limitations by incorporating scientific literature from multiple disciplines, as well as by using communication techniques, guided by the Health Promotion Model^(23)^.

### Contributions to nursing, healthcare, and/or public policy

HIV and AIDS continue to be major health concerns, and their prevention and early treatment have been established as one of the Sustainable Development Goals. For this reason, the creation and assessment of a video as a technological resource to not only promote rapid HIV testing, but also improved understanding of the infection and a clinic with an expanded role for nursing staff via gender-sensitive care, can contribute significantly to the nursing field by demonstrating interand multidisciplinary work headed by nurses.

## CONCLUSIONS

The video “Living Without Fear” was designed and evaluated. The video contributes to the scientific evidence aimed at promoting healthy behavior in gay men. In addition, a pilot group considered the video to be an educational, appropriate, and effective tool for incentivizing a culture of screening and the use of rapid tests to identify HIV infection at early stages.

The content of the video was assessed by judges and young members of the LGBTIQ+ community. These individuals considered the concepts of the Nola J. Pender Health Promotion Model, as well as the scientific evidence concerning barriers to HIV testing and the best reported strategies and audiovisual techniques. The results obtained add to the limited understanding of the creation of technology-based educational tools to promote rapid HIV testing in gay men in Mexico and Latin America from a theoretical focus on nursing.
