# Supplementary material for: Evaluation of a video to promote HIV testing in sexual minorities
Source: Rev Bras Enferm. 2024 Nov 22;77(4):e20230320. doi: 10.1590/0034-7167-2023-0320 (PMC11654221; doi:10.1590/0034-7167-2023-0320)
Supplement: Translation

# Translation: Avaliação de um vídeo para promover a testagem de HIV em minorias sexuais

**DOI:** 10.1590/0034-7167-2023-0320pt

**Authors:** Francisco Javier Báez Hernández, Vianet Nava Navarro, Miguel Angel Zenteno López, Víctor Manuel Blanco Álvarez, Arelia Morales, Pedro Trejo Hernández

**Affiliations:** I Benemérita Universidad Autónoma de Puebla. Puebla, México

**Keywords:** Filme e Vídeo Educativo, Estudo de avaliação, Recursos audiovisuais, Teste de HIV, Minorias Sexuais e de Gênero

## Abstract

**Objetivo:**

Elaborar e Avaliação um vídeo educativo voltado para a promoção da testagem anti-HIV em homens homossexuais sob a perspectiva teórica do Modelo de Promoção da Saúde de Nola J. Pender.

**Métodos:**

O desenho constou de cinco etapas: 1.- Pesquisa bibliográfica; 2.- Elaboração do objetivo educacional; 3.- Construção do guião e localização da informação nas componentes teóricas; 4.- Produção; 5.- Avaliação por especialistas e população-alvo.

**Resultados:**

Foi produzido o vídeo “Viver sem Medo”, que apresenta o dilema que um homem homossexual tem, antes de realizar o teste de HIV. O índice de avaliação de conteúdo obteve valor de 0,85, o que indica que as informações são adequadas e aceitáveis, para a promoção do teste rápido anti-HIV.

**Considerações finais:**

Os resultados contribuem para a evidência científica direcionada à promoção de comportamentos saudáveis, além de se mostrar uma ferramenta educacional aceitável.

## INTRODUÇÃO

O vírus da imunodeficiência humana (HIV) é um lentivírus da família Retroviridae que afeta diretamente os linfócitos CD4+, isto é, as células coordenadoras dos mecanismos de defesa dos seres humanos^(1)^. Em 2021, 38,4 milhões de pessoas em todo o mundo viviam com o HIV^(2)^. Estima-se que 341.313 pessoas tenham HIV no México^(3)^. A situação é semelhante no estado mexicano de Puebla, com 521 casos registrados em 2022; a população mais afetada foi a de homens gays^(4)^.

Os homens gays estão frequentemente sujeitos à discriminação e ao estigma^(5-7)^, associados a barreiras estruturais que se tornam evidentes na organização das instituições de saúde e no atendimento recebido^(8-9)^. Essa situação agrava as preocupações adicionais com a privacidade, baixo risco percebido de contrair o HIV, falta de compreensão da saúde sexual e incapacidade de encontrar locais para a realização de testes rápidos de HIV^(10-12)^.

Nesse sentido, segundo a literatura científica, os homens gays que têm o hábito de fazer o teste rápido de HIV melhoram o comportamento sexual, devido ao aumento da autoeficácia; assim, ao submeterem-se a essa triagem, apresentam melhor autoavaliação^(13-15)^.

As estratégias usadas para promover o teste de HIV incluem vídeos educativos^(16)^, caracterizados por conteúdos dramáticos e baseados em documentários que refletem as experiências reais vividas por homens gays. Esses vídeos fundamentam-se em mais de uma teoria de mudança, têm duração de 1 a 83 minutos e apresentam efeitos favoráveis de curto prazo sobre as barreiras percebidas e comportamento de risco, além de melhorarem a compreensão, a autoeficácia e a aceitação do teste de HIV na população em questão^(16-19)^.

O design e a avaliação dos vídeos educativos desenvolvidos para prevenir infecções sexualmente transmissíveis foram considerados importantes para fornecer evidências científicas, bem como para garantir a consistência do conteúdo, a aceitabilidade e a provável eficácia na população-alvo^(20-22)^.

Por esse motivo, o design e a avaliação de um vídeo com base na teoria do Modelo de Promoção da Saúde^(23)^ tornam-se relevantes. Essa teoria considera o comportamento de saúde como o resultado de processos cognitivos de atenção, retenção, reprodução e motivação, que estão associados ao aumento da aprendizagem e das habilidades relacionadas a ações preventivas. Nesse modelo, as características e experiências individuais são sempre centrais. Isso fica evidente quando se considera a criação de um vídeo enquanto ferramenta educacional que pode aumentar a conscientização sobre a importância do rastreamento como método de autocuidado e que considera as necessidades e expectativas dos homens gays.

Dessa forma, este estudo encontra justificativa na escassez de evidências sobre a temática no México, com apenas quatro vídeos promovendo o teste de HIV na página oficial do Centro Nacional de Prevenção e Controle do HIV e da AIDS^(24)^.

## OBJETIVO

Projetar e avaliar um vídeo educativo destinado a promover o teste de HIV em homens gays sob a perspectiva teórica do Modelo de Promoção da Saúde de Nola J. Pender^(23)^.

## MÉTODOS

### Aspectos éticos

Este estudo aderiu aos princípios nacionais e internacionais de bioética e à Lei de Proteção de Dados Pessoais Mantidos por Partes Privadas^(25)^ e à Lei Geral de Saúde do México^(26)^. O consentimento informado foi obtido de todos os participantes, e o estudo foi registrado em um comitê de bioética e pesquisa (SIEP/046/2021).

### Tipo de estudo

O desenho do estudo compreendeu cinco etapas baseadas em trabalhos semelhantes^(20-22,27)^, resumidas em: 1 - Revisão da literatura; 2 - Formulação do objetivo do vídeo educativo; 3 - Elaboração do roteiro e identificação das informações em função dos conceitos teóricos do Modelo de Promoção da Saúde^(23)^; 4 - Produção; e 5 - Avaliação do conteúdo por especialistas e pela população-alvo.

### Etapas do estudo

A primeira etapa compreendeu uma revisão integrativa da literatura, seguindo as cinco etapas propostas por Whittemore (2005)^(28)^: 1 - Identificação do problema; 2 - Busca na literatura; 3 - Avaliação da qualidade do estudo; 4 - Análise dos dados; e 5 - Apresentação dos resultados. A pergunta feita foi: “Quais são as melhores evidências disponíveis sobre as intervenções usadas para promover o teste rápido de HIV em homens gays?” Foram aplicados os seguintes critérios de inclusão: ensaios clínicos randomizados em inglês, espanhol ou português, sem restrição quanto ao ano de publicação e somente artigos com texto completo. Foram pesquisados dois bancos de dados científicos, PubMed e ProQuest, usando os Descritores em Ciências da Saúde (DeCS) e operadores booleanos (AND, OR, NOT) para construir cadeias de pesquisa (“intervention” OR “random clinical trial” OR “clinical trial” AND “hiv test” AND “msm” OR “gay” NOT “women”). Os critérios de exclusão foram os seguintes: metanálises ou metassínteses, literatura cinzenta, opiniões, editoriais e casos ou relatórios clínicos. Os estudos duplicados foram eliminados do banco de dados.

Além de usar o software de gerenciamento de referências Mendeley, construímos uma matriz de análise de dados contendo aqueles relacionados ao objetivo, à teoria, à população, ao método, às características da intervenção, aos resultados e às referências para identificar as estratégias e técnicas cinematográficas com os melhores resultados.

Na segunda etapa, formulamos o objetivo do vídeo educacional para melhor conceituar e planejar a criação do produto audiovisual. Essa etapa nos ajudou a definir o tipo de vídeo educativo, bem como os elementos mais adequados à sua finalidade e às características do público-alvo (faixa etária, mídia e viabilidade de execução). Para sua criação, usamos a taxonomia de Benjamin Bloom^(29)^, seguida de uma descrição da área de realização (conteúdo ou tópico), do indicador (critério) e das estratégias ou considerações para alcançá-la.

A terceira etapa foi dividida em duas fases. A primeira envolveu a elaboração do roteiro com o apoio de uma equipe multidisciplinar. A equipe incluiu especialistas em comunicação social, escolhidos em razão de sua experiência na produção de vídeos relacionados à saúde, e especialistas no atendimento de pessoas diagnosticadas com HIV. Todos foram convidados a participar por e-mail. A elaboração do roteiro seguiu o que foi relatado em estudos relacionados e informações contidas no Centro Nacional Mexicano para a Prevenção e Controle do HIV e da AIDS e nas sugestões de um grupo de jovens homossexuais masculinos autoidentificados como líderes comunitários. Primeiro, foi explicado a esses jovens o objetivo da criação do vídeo para motivar sua participação. Então foram convidados e selecionados de forma conjunta por um recrutador de participantes-chave e pelos pesquisadores.

A segunda fase compreendeu relacionar as informações do roteiro em função dos conceitos teóricos do Modelo de Promoção da Saúde^(23)^. Isso foi feito por meio da criação de uma matriz que relacionava os conceitos teóricos do Modelo de Promoção da Saúde aos componentes do vídeo educativo (Conteúdo, Imagens e Texto)^(22)^.

A quarta etapa foi a produção do vídeo educativo, transcorrendo em um prédio público com a pré-aprovação do órgão responsável. A maioria dos atores, inclusive o personagem principal, eram membros da comunidade LGBTIQ+. O áudio foi gravado em um local silencioso com acústica adequada. A edição foi realizada com o software CapCut; e a versão inicial foi convertida para o formato MP4.

Finalmente, a quinta etapa compreendeu a avaliação do conteúdo por seis pesquisadores considerados especialistas em virtude de sua carreira e publicações sobre saúde sexual e HIV. Os pesquisadores avaliaram os seguintes aspectos: objetivo (focado em cuidados, desmistificação, prevenção, informações e ideias sobre o HIV), linguagem (clareza da mensagem), relevância (eficácia, adequação e consistência do vídeo), conteúdo (história clara, objetiva e cientificamente precisa baseada em evidências) e design (apresentação atraente e duração adequada)^(30)^. Uma escala Likert foi usada para pontuar esses aspectos de 1 a 5 (1 = discordo totalmente; 2 = discordo; 3 = neutro; 4 = concordo; 5 = concordo totalmente). Os dados foram processados no Microsoft Excel, usado para obter o índice de validade de conteúdo (IVC)^(31)^. O conteúdo do instrumento foi considerado adequado com uma pontuação de IVC igual ou superior a 0,80.

### Análise dos resultados e estatísticas

Além disso, aplicamos pesquisas de aceitabilidade e eficácia preliminar^(18)^, adaptadas para este estudo em um grupo de 18 homens gays. Dois recrutadores de participantes selecionaram esses indivíduos que pertenciam à comunidade LGBTIQ+ e iniciaram a amostragem em bola de neve. Os participantes tiveram uma semana para responder às seguintes perguntas: “O quanto você gostou do vídeo Viver sem Medo?; O quanto você recomendaria o vídeo Viver sem Medo a um amigo?; Às vezes, foi difícil entender o que estava acontecendo no vídeo?; Às vezes, sua mente ficou divagando enquanto assistia ao vídeo?; Os eventos da história retratada no vídeo foram relevantes para sua vida diária?; Você acha que as informações fornecidas sobre determinados tópicos foram imprecisas ou enganosas?; Você entendeu os sentimentos e as emoções do personagem principal?; No futuro, você planeja fazer o teste de HIV regularmente?; e Você gostaria de marcar uma consulta para fazer o teste de HIV?”. A avaliação foi satisfatória, com a maioria das respostas indicando boa compreensão e aceitabilidade do conteúdo do vídeo. Todas essas análises foram realizadas usando o programa Statistical Package for the Social Sciences (SPSS versão 26.0), que forneceu as frequências e porcentagens de cada pergunta.

## RESULTADOS

A revisão da literatura identificou estudos randomizados com as seguintes características: uso de redes eletrônicas de mídia social, páginas da web e vídeos. Esses últimos eram geralmente curtos, forneciam informações simples sobre o HIV e promoviam o teste. Além disso, eles usaram teorias comportamentais, como a pesquisa participativa baseada na comunidade, a estrutura cognitiva social, o modelo de crença na saúde e o modelo de comportamento integrado. As estratégias usadas abrangeram a inclusão de colegas e líderes comunitários, bem como atores habituados com o uso do vernáculo familiar à população-alvo. Além disso, foram consideradas as barreiras socioculturais e econômicas, bem como os motivos e as experiências significativas dos participantes, não apenas quando eles estão fazendo o teste rápido de HIV, mas também quando são sexualmente ativos.

Após a análise da literatura, o objetivo geral foi estabelecido selecionando-se um verbo de ação do domínio afetivo da taxonomia de Bloom^(29)^. Esse domínio se concentra nos comportamentos observáveis dos indivíduos, incluindo o aprendizado associado a sentimentos, emoções e atitudes. A finalidade era motivar os homens gays a fazer o teste rápido de HIV, considerando suas percepções, influências, crenças e experiências que favorecem ou limitam o comportamento de promoção da saúde (Quadro 1).

**Quadro 1:** Objetivo do vídeo educativo

*Promover o teste rápido de HIV em homens jovens em Puebla por meio do testemunho narrativo de um caso bem-sucedido e sob a perspectiva teórica de Murdaugh, Parsons e Pender (2018).*

Com base no ponto anterior, desenvolvemos o roteiro de um vídeo intitulado “Vivir sin Miedo” (Viver sem Medo). O roteiro considerou as opiniões de um grupo de jovens homossexuais, bem como certas ferramentas de programação neurolinguística e marketing sensorial, uma vez que a percepção é influenciada por crenças e valores. Por esse motivo, a comunicação não se limita à linguagem escrita ou falada: envolve várias ferramentas relacionadas a sentimentos e lembranças que estão embutidas na mente consciente ou inconsciente de um indivíduo e que podem ajudar a fortalecer a mensagem^(32-33)^. Em conjunto, a eficácia da recepção de informações aumenta com o acesso a todos os sentidos, permitindo a persuasão mais completa e interativa dos indivíduos^(34-35)^.

Diante do exposto, decidimos usar o testemunho narrativo, uma técnica de comunicação que se apoia em uma abordagem fenomenológica para apreciar experiências. Essa técnica pondera os fatos com base em como eles são vividos e sentidos e como influenciam e afetam os indivíduos, facilitando assim a compreensão de valores, situações, história e experiências vividas^(36)^. Uma vez obtido o roteiro, essa abordagem nos permitiu relacionar todas as informações em função dos conceitos do Modelo de Promoção da Saúde^(23)^ (Quadro 2).

**Quadro 2:** Relação do Modelo de Promoção da Saúde com os componentes do vídeo educativo

Modelo de promoção da saúde	Conteúdo	Imagem	Texto
-----	Créditos de abertura	Título do vídeo	“Vivendo sem medo”
Comportamento de promoção da saúde	Vírus que ataca as defesas do corpo e pode ser controlado.	Eduardo olha para a tela do dispositivo que lhe dará o resultado do teste. Ele olha para a câmera.	Meu nome é Eduardo e, há alguns dias, tive uma experiência que me mudou para sempre.
Influências situacionais	Por contato com fluidos corporais, debido a sexo desprotegido e compartilhamento de agulhas infectadas. Às vezes, durante a gravidez, parto ou amamentação.	Eduardo recebe mensagens de WhatsApp de seu parceiro sexual descrevendo os sinais e sintomas da infecção pelo HIV.	Depois de estar com Gabo, as mensagens que ele me enviou são aquelas que ninguém quer receber. Ele estava passando por um momento difícil.
Barreiras percebidas para a ação	Ela não é transmitida por picadas de insetos, saliva, lágrimas ou suor, nem por abraços, mãos dadas, uso do mesmo banheiro, compartilhamento de pratos ou “beijos sociais”.	Eduardo está claramente ansioso e não consegue dormir em seu quarto.	Algo estava errado, e isso não parava de rondar minha cabeça. Fala-se tanto sobre o HIV que não sei em que acreditar: se é possível contrai-lo usando o mesmo banheiro ou compartilhando pratos. Minha cabeça vai explodir!
Influências situacionais	Os mitos do HIV (p.ex., o de que somente determinados grupos podem contrair o vírus) prejudicam qualquer pessoa que se submeta ao teste e sugerem que se trata de alguma forma de punição divina.	Eduardo, verificando o Grindr, encontra o anúncio de testes rápidos na BUAP Clinic.	Somente nós entendemos e apoiamos uns aos outros. O que é isso? A BUAP realiza testes rápidos, e o centro fica perto daqui! Hmmm… mas não sei. Há tantos odiadores, e eu não consigo lidar com isso.
Autoeficácia Fatores socioculturais pessoais	O teste rápido é eficaz se for realizado entre 18 e 90 dias após a exposição.	Eduardo decide comparecer ao centro para fazer o teste. Eduardo encontra uma atmosfera calorosa e amigável (homens e mulheres educados). Uma garota o leva ao consultório. Um casal sorridente deixa o consultório.	Já me decidi: se eu não cuidar de mim, como poderei cuidar do Gabo? Que lugar legal. Ninguém está me dando más vibrações. Nossa, eles são gente como eu. Amigáveis. Aqui vou eu.
Influências situacionais Comportamento de promoção da saúde	Seu dedo é picado para coletar uma gota de sangue, a qual será posta em contato com um reagente, revelando o resultado positivo ou negativo.	Eduardo entra na sala de consulta e é recebido por um profissional de saúde Eduardo se submete ao teste.	A equipe de enfermagem que me atende é muito discreta, limpa e descontraída. Hmmm… um pouco assustador, mas, por favor, é apenas um idiota.
Barreiras percebidas para a ação Comportamento de promoção da saúde Compromisso com o plano de ação	Você tem o resultado em menos de dez minutos. Ele é 98% preciso. Não é necessário fazer jejum antes. A consulta é discreta, anônima e confidencial. O resultado lhe dá paz de espírito, controle sobre sua saúde e confiança para conduzir sua vida sexual de forma plena e responsável.	O resultado do teste de Eduardo é negativo e ele recebe mais informações (sobre o teste e HIV). Transição Eduardo acompanha seu parceiro para fazer o teste na clínica.	Que legal! Em dez minutos, todas as dúvidas foram sanadas: eles explicaram que o resultado é 98% preciso e que não é necessário nenhum preparo. É discreto, anônimo e confidencial. Agora posso desfrutar plenamente de minha vida sexual. Bem, Gabo e eu podemos. Isso é amar a si mesmo... vamos lá!
--------	Créditos de encerramento	Créditos e logotipos	Tela preta

A produção do vídeo durou uma semana. Nesse tempo, foram adicionados os seguintes efeitos, além das legendas em espanhol: flashback, tomadas em preto e branco, transições de cores, tomadas em câmera lenta e tomadas inclinadas para baixo em direção aos olhos do ator principal. O resultado final foi um vídeo com duração de 1 minuto e 49 segundos. Posteriormente o conteúdo foi avaliado por seis especialistas em saúde sexual e HIV (médicos, equipe de enfermagem, psicólogos, antropólogos e químico farmacobiológico). Foi obtido um IVC de 0,85, indicando que as informações utilizadas eram adequadas e apropriadas para promover o teste rápido de HIV em homens gays (Tabela 1). Entretanto, consideramos primeiro as recomendações dos juízes no domínio “Objetivo”, visando tornar os fatores de risco mais explícitos, como a atividade sexual desprotegida e a criação de espaços amigáveis sem estigma relacionado à preferência sexual, que é uma das estratégias de prevenção do HIV.

**Tabela 1:** Avaliação pelos juízes

Domínio	Pergunta	CVI
1 - Objetivo	O filme faz os espectadores pensarem sobre o cuidado que os homens gays devem ter em suas relações sexuais?	0,73
	Ele ajuda a desmistificar o tema do HIV?	0,83
	Promove uma mudança de comportamento e atitude em relação à falta de prevenção?	0,83
	Esclarece as informações sobre o teste rápido de HIV?	0,70
	Diminui os preconceitos em relação ao HIV?	0,80
2 - Linguagem	O conteúdo apresentado é compreensível?	0,80
	O conteúdo é compreensível devido ao uso de palavras familiares e definições simples?	0,90
	O conteúdo é compreensível devido à boa concordância gramatical?	0,90
3 - Relevância	É relevante como uma ferramenta de educação em saúde?	0,93
	É adequado para homens gays?	0,93
	Oferece uma oportunidade para entender melhor o teste rápido de HIV?	0,90
	Ele se concentra no aspecto principal, o desempenho do teste rápido de HIV?	0,93
4 - Conteúdo	É capaz de esclarecer dúvidas sobre o teste rápido de HIV?	0,76
	As mensagens são apresentadas de forma clara e objetiva?	0,80
	As informações apresentadas são cientificamente corretas?	0,93
	O conteúdo é suficiente para atingir o objetivo?	0,70
	Há uma sequência lógica para as informações apresentadas?	0,86
5 - Design	A apresentação do vídeo é atraente e bem organizada?	0,96
	O vídeo tem a duração adequada?	0,96
Geral		0,85

*IVC - índice de validade de conteúdo.*

Por fim, o vídeo foi exibido para 18 homens gays, principalmente estudantes universitários, com idade média de 20,1 ± 1,25 anos (variação de 18 a 21 anos). As pesquisas preliminares de aceitabilidade e eficácia propostas por del Rio-González et al. (2021)^18^ revelaram uma alta porcentagem de aceitação e recomendação entre as pessoas que assistiram ao vídeo “Living without Fear”.

Em relação ao engajamento narrativo, a maioria dos pesquisados achou fácil entender o que havia acontecido no vídeo e sentiu que os eventos da história eram relevantes para suas vidas. Para a contra-argumentação, a maioria dos pesquisados considerou que as informações mostradas no vídeo eram precisas e confiáveis e se identificou com os sentimentos ou emoções do personagem principal. Quanto ao teste de HIV, todos os entrevistados afirmaram que, depois de assistir ao vídeo, planejavam fazer o teste (Tabela 2).

**Tabela 2:** Aceitabilidade e eficácia do vídeo “Viver sem Medo”

Dimensão	Pergunta	Tipo de resposta							
		Um pouco		Mais ou menos		Muito		Muitíssimo	
		%	n	%	n	%	n	%	n
Aceitabilidade	O quanto você gostou do vídeo “Viver sem Medo”?	5,6	1	33,3	6	33,3	6	27,8	5
	O quanto você recomendaria o vídeo “Viver sem Medo” a um amigo?	0,0	0,0	27,8	5	38,9	7	33,3	6
**Dimensão**	**Pergunta**	**Nada**		**Um pouco**		**Muito**		**Muitíssimo**	
		**%**	**n**	**%**	**n**	**%**	**n**	**%**	**n**
Contra-argumentação	Você acha que as informações fornecidas sobre determinados tópicos eram imprecisas ou enganosas?	61,1	11	38,9	7	0,0	0	0,0	0
Identificação com o personagem principal	Você entendeu os sentimentos e as emoções do personagem principal?	0,0	0	5,5	1	66,7	12	27,8	5
**Dimensão**	**Pergunta**	**Discordo totalmente**		**Discordo**		**Concordo**		**Concordo totalmente**	
		**%**	**n**	**%**	**n**	**%**	**n**	**%**	**n**
Envolvimento com a narrativa	Às vezes, foi difícil entender o que estava acontecendo no vídeo?	44,5	8	33,3	6	22,2	4	0,0	0
	Às vezes, sua mente se distraiu enquanto assistia ao vídeo?	50,0	9	50,0	9	0,0	0	0,0	0
	Os eventos da história retratados no vídeo foram relevantes para sua vida diária?	0,0	0	16,7	3	55,6	10	27,7	5
Intenção de fazer o teste de HIV	No futuro, você planeja fazer o teste de HIV regularmente?	0,0	0	0,0	0	38,9	7	61,1	11
	Você gostaria de marcar uma consulta para fazer um teste de HIV?	0,0	0	11,2	2	44,4	8	44,4	8

*n - número de participantes; % - porcentagem.*

## DISCUSSÃO

O presente estudo relatou o processo de concepção e avaliação de um vídeo educativo destinado a promover o teste de HIV em homens gays. Essa ação de triagem reflete um estado de conscientização após a exposição ao risco, incentivando tanto o autocuidado quanto o cuidado com os outros em caso de infecção, sob a perspectiva teórica do Modelo de Promoção da Saúde^(23)^ Nossa abordagem foi baseada em uma análise da literatura científica, semelhante à realizada por outros estudos^(20,22)^, nos quais se mostrou que os recursos audiovisuais (vídeos) são uma ferramenta promissora para a promoção do comportamento sexual saudável. Esse vídeo pode ter efeitos positivos importantes sobre os homens gays mexicanos se for divulgado nos aplicativos de encontros gays.

O objetivo do vídeo “Living without Fear” (Viver sem Medo) foi estabelecido de maneira semelhante à realizada no Brasil^(27)^, em que foi usado o método CTM3. Primeiro conceituamos o produto que desejávamos obter. Isso nos permitiu refletir sobre o que Bloom menciona em sua teoria de aprendizagem^(29)^, especificamente acerca das características afetivas de entrada que motivam os indivíduos. Essas características podem levá-los a alcançar resultados de aprendizagem e, portanto, potencialmente inspirar mudanças em atitudes e comportamentos relacionados ao autocuidado entre homens gays.

A elaboração do roteiro e a produção do vídeo foram novamente semelhantes às de outros estudos realizados no Brasil^(21-22)^. Durante todo o processo, consideramos as opiniões e as experiências vividas pelos homens gays (em linha com outros estudos do México^(5)^ e do Brasil^(12)^), bem como os preconceitos relacionados às necessidades de saúde dos homens gays, uma situação que desencadeia o medo e a autoexclusão do atendimento médico.

Essa última reflexão nos permitiu incorporar ao roteiro a ferramenta de programação neurolinguística “ancoragem visual”^(32)^, a qual foi associada à experiência emocional de homens gays que fizeram o teste de HIV pela primeira vez. Essa ferramenta foi aprimorada por estratégias de marketing sensorial, que foram elementos-chave na narrativa do depoimento. Tais estratégias presumiram que a experiência é uma unidade significativa e deram voz àqueles que foram excluídos, tornados invisíveis e marginalizados pela sociedade^(36)^.

Devido aos elementos contidos no roteiro, o vídeo “Living without Fear” se alinha com o conceito de Influências Interpessoais dentro dos conceitos teóricos do Modelo de Promoção da Saúde^(23)^. Isso porque se trata de uma fonte primária cuja finalidade é convencer homens gays a adotarem comportamentos de saúde sob o pressuposto de que os indivíduos, em toda a sua complexidade biopsicossocial, podem mudar progressivamente ao longo do tempo.

Nesse caso em particular, operacionalizamos o aspecto acima no *storyboard* por meio da inter-relação de fatores socioculturais pessoais, influências situacionais, benefícios, barreiras e autoeficácia percebida, usando tanto atores pertencentes à comunidade LGTBQ+ quanto linguagem e locais reconhecidos pela população-alvo. Além disso, o vídeo mostrou o conflito que eles enfrentam ao considerar o teste rápido de HIV e como o superam, ou seja, encontrando um local acessível, discreto e respeitoso. Em conjunto, esses fatores geram um compromisso com um plano de ação e, por conseguinte, um comportamento de promoção da saúde, especificamente a aceitação e a realização do teste rápido de HIV. Essa situação atua não apenas como base para as ações de cuidado, mas também gera uma ponte entre a teoria e a Prática Avançada de Enfermagem, localizada no nível de atenção primária.

Quanto ao processo de avaliação, os achados estão de acordo com os de outros estudos que abordam tanto questões de doenças sexualmente transmissíveis no trabalho social^(30)^ quanto a promoção da vacinação contra o HPV^(21-22)^ com pontuações adequadas nos domínios de linguagem, relevância, conteúdo e design. Os resultados mostram que o vídeo “Viver sem Medo” é uma ferramenta educacional aceita e recomendada para a prevenção e promoção do teste rápido de HIV. Além disso, a proposta foi enriquecida e aprimorada pelas opiniões dos juízes para atingir o objetivo proposto.

Quanto às pesquisas de aceitabilidade e eficácia, os resultados estão de acordo com os obtidos na Colômbia^(18)^, que mostraram boa aceitabilidade pela população de homens gays, principalmente por meio da autoidentificação com o personagem principal e sua contra argumentação e intenção de fazer o teste de HIV. No entanto, os resultados do engajamento narrativo foram maiores em nosso vídeo. A razão disso pode estar na curta duração do filme bem como na incorporação das ações verbais e não verbais e da linguagem do personagem principal, que poderiam ter sido identificadas como parte da vida cotidiana da população-chave. Esse resultado destaca a importância de sempre considerar a participação e as opiniões dos homens gays ao elaborar materiais educativos sobre saúde.

### Limitações do estudo

Como limitações, observamos que o vídeo foi feito com base nas percepções e gostos de um grupo de homens gays da cidade de Puebla. Dessa forma, os resultados não podem ser generalizados para toda a comunidade LGTBQ+ no México porque os gostos mudam constantemente e são limitados à cultura e ao contexto social onde o estudo foi realizado. Além disso, o conhecimento sobre o HIV não é estático, logo é necessária uma atualização contínua das informações fornecidas. Todavia, buscamos reduzir essas limitações incorporando a literatura científica de várias disciplinas e usando técnicas de comunicação, orientadas pelo Modelo de Promoção da Saúde^(23)^.

### Contribuições para a enfermagem, saúde e/ou políticas públicas

O HIV e a aids continuam a ser grandes preocupações de saúde, de modo que sua prevenção e tratamento precoce foram estabelecidos como um dos Objetivos de Desenvolvimento Sustentável. Por esse motivo, a criação e a avaliação de um vídeo como recurso tecnológico para promover não apenas o teste rápido de HIV, mas também uma melhor compreensão da infecção e uma clínica com papel ampliado para a equipe de enfermagem por meio de cuidados sensíveis ao gênero, podem contribuir significativamente para o campo da enfermagem, demonstrando o trabalho interdisciplinar e multidisciplinar liderado por enfermeiros.

## CONCLUSÕES

O vídeo “Living without Fear” foi projetado e avaliado. Ele contribui para as evidências científicas destinadas a promover um comportamento saudável em homens homossexuais. Além disso, um grupo-piloto considerou o vídeo uma ferramenta educativa adequada e eficaz para incentivar uma cultura de triagem e o uso de testes rápidos voltados a identificar a infecção pelo HIV em estágios iniciais.

O conteúdo do vídeo foi avaliado por juízes e jovens membros da comunidade LGBTIQ+. Eles consideraram os conceitos do Modelo de Promoção da Saúde de Nola J. Pender, evidências científicas sobre as barreiras ao teste de HIV e as melhores estratégias e técnicas audiovisuais relatadas. Os resultados obtidos contribuem para a compreensão limitada da criação de ferramentas educacionais baseadas em tecnologia para promover o teste rápido de HIV em homens gays no México e na América Latina, sob um enfoque teórico em enfermagem.
